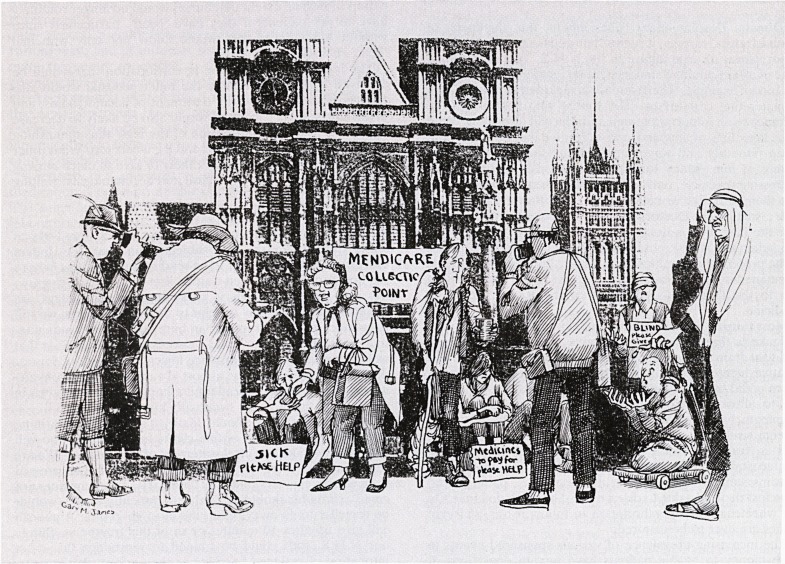# New Dimensions in the N.H.S.

**Published:** 1989-02

**Authors:** J. L. Burton

**Affiliations:** Consultant Dermatologist, Department of Dermatology, Bristol Royal Infirmary, BS2 8HW


### New Dimensions in the NHS

**J. L. Burton**
Consultant Dermatologist, Department of Dermatology, Bristol Royal Infirmary, BS2 8HW.

Travel, they say, adds a new dimension, and the traveller in India is guaranteed a truly 4D experience, characterized by frequent encounters with Death, Disease, Defaecation and Delay. These subjects are not unfamiliar of course to those of use who labour at the sharp end of the National Health Service, but our problems are small compared with those faced by Indian health officials. Is it possible that the N.H.S. might have something to learn from the way in which India tackles its 4D problems?

*1. Death.* The sight of a dying person on a crowded pavement is commonplace in urban India, and parts of some cities such as Calcutta can almost be regarded as an enormous hospice for the dying, with giant outdoor wards. Surely our political leaders must realize that there is scope for vast savings to be made in the N.H.S. in this area of health care.

Admittedly we have already made a start in the right direction by discharging so many of our chronic psychiatric patients into the community. Their tendency to congregate under Vauxhall Bridge, sleeping in large cardboard boxes which they have themselves provided, shows the enormous potential of this idea. Many of our bridges throughout the country are underused at present and most supermarkets have surplus cardboard boxes. Tremendous savings could be made by persuading the dying to join the socially inadequate in utilizing these existing facilities to the full. It should be possible to calculate the amount by which the top level of income tax could be further reduced if such a policy of 'Outdoor Inner City Hospices' were to be introduced throughout the country.

There would probably be some initial opposition to this scheme, based on political rather than economic grounds, but the key to the success of this social experiment would be to persuade the British people to embrace the Hindu religion, particularly the philosophy of 'dharma' and 'karma'. Current trends in the N.H.S. suggest that doctors and nurses will themselves lead the way in this cultural revolution.

'Dharma' refers to the inescapable acceptance of things as they are, in the knowledge that things are pre-ordained and it is hopeless to try and change them. The only sensible thing to do is to accept one's lot as gracefully as possible. Considerable progress has been made in inculcating this philosophy into senior N.H.S. staff in the last few years, and most Consultants and Ward Sisters now have a firm grasp of the concept of 'dharma'.

'Karma' refers to the idea that if one accepts one's station and performs one's duties stoically in this life, one can expect to be granted higher status in a future life, following reincarnation. This philosphy too is slowly becoming accepted by

N.H.S. staff, and most Surgical Senior Registrars now accept that this is their best hope for the future.

The corollary of the 'karma' idea is that consultants who do not accept their 'dharma' gracefully but try to improve their status by various petty acts of self-aggrandizement are likely to end up as earthworms in the next life. This too is likely to prove a popular concept in many quarters in the N.H.S.

2. *Disease.* Despite many jibes about the thickness of administrators' carpets, it seems that disease is still one of the major causes of expenditure in the N.H.S. This unfortunate state of affairs could to some extent be rectified if we followed the Indian example, where disease is regarded as a useful way of generating cash-inflow. The tourist who approaches the entrance to an important temple in India will often be greeted by a long line of maimed and deformed beggars who will moan piteously and wave their various sores and festering stumps at him. Many tourists try studiously to ignore this distressing spectacle, only to find that the beggars hop directly into their path, like wounded crows, until they are bought off with a small coin. Doctors who are seasoned travellers know that the best ploy is not to give them money (which is like giving jam to a wasp), but to take a keen professional interest in the proferred clinical lesion. I usually find that the variants of cutaneous tuberculosis alone provide better value than a trip to the Dermatology Section of the Royal Society of Medicine.

Most tourists are not doctors though, and the point I wish to make is that disease and disability need not always be a financial drain on the community, but should be regarded as a positive asset which can be turned into hard cash. Surely space could be found outside Westminster Abbey for example to allow suitable patients to improve our balance of payments by wringing dollars, marks and yen from our foreign visitors.

It is true that some tourist venues, the Huddersfield Pentecostal Church for example, lack the cachet of Westminster Abbey, but even in these areas, patients who have lost their jobs due to disease could be reminded that 'On yer wheelchair' is the exhortation to follow if our Victorian values are ever to be restored.

The increasing prevalence of various sponsored events to raise money to enable patients have private operations or special items of equipment shows that the concept of 'Charity-dependent treatment' is not entirely alien to the British public.

3. *Defaecation.* A great deal of nurses' time is spent in British hospitals in enquiring about patients' bowel functions, and this is necessitated by the British obsession with the importance of privacy during defaecation. Most Indians of course do not share this obsession, and evidence of their lack of fastidiousness in this regard is to be found throughout India, particularly on river-banks -and pavements. It seems unlikely that this relaxed approach to 'squatters' rights' will ever become acceptable in Britain, if only for public health reasons, but surely we could lose our inhibitions to the extent that useful economies could be made throughout the N.H.S. by dispensing with toilet doors.

But why stop there? Should we not be a healthier race if we dispensed with the toilet pedestal too? If a hole in the floor is good enough for the public toilets in India, why do British hospitals need to waste money on unnecessary porcelain? The squatting position is undoubtedly an aid to satisfactory defaecation, and would be likely to reduce the incidence of constipation to such an extent that nurses would no longer routinely have to ask patients if they have 'been', particularly since without toilet doors they would know not only who had 'gone', but where and when.

The concomitant reduction in constipation that would be achieved by the abolition of the toilet pedestal would also reduce the need for surgical treatment of haemorrhoids, and according to some experts, it would also be likely to decrease the risk of venous hypertension of the legs, thus diminshing the incidence of varicose veins and leg ulcers too. What other saving in capital expenditure is likely to save so much nursing time, as well as reducing surgical and dermatological waiting lists?

4. *Delay.* Some might argue that the N.H.S. has little to learn from India on this subject, and that the Indian Government should really to be sending study groups here to see how properly nurtured waiting lists can be made to grow. My own feeling though is that although our delays are quantitatively in no way inferior to the Indian efforts, we still have much to learn from Indian bureaucracy from a qualitative point of view. Our main weakness in the N.H.S. is that we are forever trying to produce logical explanations for our delays. Not only are these almost always entirely unconvincing, but they tend to engender false hopes that somebody is in control and things might eventually improve.

Our administrators should learn to adopt the disarming approach of the Indian airline clerk, for example, who will courteously make a neutral statement of fact, such as 'We are regretting that there will be no planes today'. When pressed for a reason, he will simply say 'Some days the plane does not come', shrug his shoulders, and wait patiently for the would-be traveller to go away. Even the most go-getting American will give up after 10 minutes or so of this treatment. Surely our N.H.S. staff could be trained to reproduce this exact intonation of resigned passivity as they inform the exasperated patient that 'Some days the ward is closed', 'Some days there are no blankets' etc.

Admittedly this training will take time, but there are encouraging signs that we have made some progress in this area. If you don't believe me try to find out why the white coats are often late back from the laundry.

It will be clear from these few suggestions that major savings could still be made in several areas of health care if we could only agree to follow the Indian example of self-sufficiency. For this health care policy to be truly successful however it is vital that we should continue to pursue the prudent policies of enlightened self-interest which were so dear to our fore-fathers, and thus enable the rich to gain satisfaction in this life, and preferment in the next, by countless small acts of charity.

## Figures and Tables

**Figure f1:**